# Impact of the Covid-19 pandemic and ensuing online teaching on pre-clinical medical education

**DOI:** 10.1186/s12909-023-04967-x

**Published:** 2024-01-17

**Authors:** Houman Goudarzi, Masahiro Onozawa, Makoto Takahashi

**Affiliations:** 1 Center for Medical Education and International Relations, Faculty of Medicine, Graduate School of Medicine, Hokkaido University, Sapporo, Japan; 2 Clinical Training Center, Hokkaido University Hospital, Sapporo, Japan

**Keywords:** COVID-19 pandemic, Medical education, Medical students, Preclinical clerkship, Online and in-person teaching, Subjective and objective assessments, Academic performance

## Abstract

**Background:**

Major disruptions and changes in education have occurred worldwide as a result of the coronavirus disease (COVID-19) pandemic and the ensuing shift from in-person to online education. However, the effect of such changes on medical education, its magnitude, and the learning domains impacted by such rapid changes have not been adequately addressed, particularly with regard to objective assessment approaches.

**Methods:**

Second-year medical students enrolled in our Medical English Course between 2019 and 2021 were recruited from Hokkaido University, Japan (*N* = 321) to participate in this study. We evaluated the potential impact of teaching styles on the academic performance of students before (2019; face-to-face) and during (2020; online; 2021; in-person and online) the pandemic. We examined the potential effect of three teaching styles––in-person (2019), online (2020), and a combination of these (2021) on the academic performance of medical students using: *(i)* subjective assessment of self-reported general English skills, including reading, writing, listening, and speaking; and *(ii)* objective assessment of medical terminology scores, evidence-based medicine (EBM) skills, and final written exam scores.

**Results:**

In-person education significantly improved listening and speaking skills in 2019 (*p* < 0.001). This trend was observed for writing skills in an online course in 2020 (*p* = 0.001). With the combined teaching method, students reported significant improvements in all four English skills. In our objective assessments, medical terminology improved significantly post-test versus pre-test for all three teaching styles, and we found that the online course did not adversely affect the gain in medical terminology knowledge during the course. Additionally, we did not find any significant differences across the three applied teaching styles regarding EBM skill levels. It is noteworthy that the students taking online courses had a significantly higher final exam score (mean ± SD; 82.8 ± 8.2) than in in-person (78.6 ± 8.8) and combined (79.7 ± 12.1) teaching styles.

**Conclusions:**

In our study, the online/combined courses showed better academic outcomes compared to the face-to-face course in the preclinical clerkship. Although the current results need to be replicated on a larger scale, online/combined courses can continue and evolve in the post-pandemic education of medical students. Medical schools and institutions should consider incorporating such courses, especially combined courses, into their curricula in the future to improve the effectiveness, accessibility, and flexibility of medical education.

**Supplementary Information:**

The online version contains supplementary material available at 10.1186/s12909-023-04967-x.

## Background

 The coronavirus disease (COVID-19) pandemic has disrupted education in medical schools, hospitals, and healthcare facilities. Medical schools have implemented various preventive measures to minimize the spread of COVID-19. Therefore, such schools and educators have adapted their programs to maintain educational activities by quickly switching from in-person teaching methods to online teaching [1, 2]. Online education does not have time or space limitations, and students can take lessons at home to help prevent the spread of COVID-19. However, the efficacy of online education for medical students during the historically unprecedented COVID-19 pandemic has not been adequately explored.

Through a meta-analysis, Cook et al. [3] summarized all the studies on internet-based instruction involving upcoming health professionals, including student physicians, nurses, pharmacists, and dentists. The authors conducted two systematic reviews and meta-analyses and found that internet-based interventions had positive effects compared with no interventions. However, the effects and statistical heterogeneities were small compared with those of offline teaching. Another recent systematic review and meta-analysis restricting participants to licensed healthcare professionals suggested that online methods may be as effective as alternative methods for healthcare professionals. However, the total effect of online learning was small, without a significant difference, compared with offline teaching [4].

Some recent studies conducted during the COVID-19 pandemic on online teaching have indicated the possible influence of the pandemic on medical education; however, the results are debatable. A Polish survey [5] reported medical students’ perceptions of e-learning. They acknowledged the advantages of online courses, such as staying at home, continuous access to online materials, and learning at their own pace. However, online learning was inferior to face-to-face learning in terms of social competence and skills. A Korean study reported an overall decline in medical students’ academic performance after switching to online classes [6]. In contrast, another study suggested that the pandemic and online teaching did not influence the overall performance of Chinese medical students [7]. However, few studies have examined the effects of online versus face-to-face using subjective and objective measures in preclinical clerkships among medical students. Also, the impact of combined courses in addition to online courses has not been well studied.

Owing to the significant consequences of the COVID-19 pandemic and potentially similar consequences of future pandemics, we examined the impact of COVID-19 and online education with a synchronous distance education strategy on the academic performance of medical students through subjective and objective assessments. We applied in-person (2019), fully online (2020), and a combination of these methods (2021) to a medical English course taught to second-year medical students. We asked the students to self-assess their English skills before and after the course, using online and/or in-person learning methods for subjective assessment. We also objectively examined the impact of the teaching style on student performance, including medical terminology scores, evidence-based medicine skills, and final exam scores.

## Materials and methods

### Study participants

A total of 321 medical students, who enrolled between 2019 and 2021 at Hokkaido University, Sapporo, Japan, were included in the current analysis. We conducted an annual medical English course for second-year medical students, including a 15-session course between April and July. Our course was a bilingual (Japanese and English) one that focused on the core competencies of English for medical purposes, including doctor-patient (taking history and physical examination) and doctor–doctor (evidence-based medicine, medical terminology, scientific presentation, etc.) communication, to inspire students studying medicine in English. In 2019, all lessons were conducted face-to-face; however, the course was held entirely online in 2020 owing to the COVID-19 pandemic. In 2021, half of the courses were held in person and half online, according to real-time restrictions regarding COVID-19 measures (For more information, see Supplementary Data, Figure S1).

### Subjective assessment

Using a questionnaire-based survey, the students self-estimated their general English skills, including reading, writing, listening, and speaking, on a Likert scale ranging from 1 (minimum) to 5 (maximum). We collected such data before and after the course to compare how our course influenced the students’ skills.

### Objective assessments

For an objective assessment, we examined the core competencies of medical English education in our course before and after the COVID-19 pandemic (in-person versus online education). The objectives of this study are in line with the Model Core Curriculum for Medical Education in Japan 2022 [8].

A) Medical terminology: We conducted pre-and post-test exams before and after medical terminology lessons using the same sets of ten multiple-choice questions (MCQs) between 2019 and 2021 to assess the improvement in students’ knowledge.
B) Evidence-based medicine: We divided the students into small groups of six or seven members (a total of 15–16 groups), held two lessons and read a paper from the *New England Journal of Medicine*. We taught them how to read, analyze, and extract the latest medical information from top medical journals. After completing the lessons, the students were asked to write a report, summarize the paper, preparing poster and present the main points of the paper orally. Evaluation of evidence-based medicine skills performed by a teaching faculty member using the same set of reports between 2019 and 2021, with ten questions, including seven MCQs and three open questions.
C) Final exam: After completing the course, the students took a final written exam with 25 MCQs.

### Ethical considerations

After explaining the aim of this study, we asked all second-year medical students in our school to participate in the first lesson of the course. No financial or other incentives were provided for their participation. The questionnaire included an opt-out item for students who did not want to be included in the data analysis. Ethical approval was obtained from the Institutional Review Board of the Faculty of Medicine and Graduate School of Medicine, Hokkaido University (20–040).

### Statistical analysis

Data were reported using descriptive statistics. Participants who preferred not to be included in the data analysis were excluded (*n* = 8). Numerical comparisons were made using one-way analysis of variance, two-tailed t-tests and paired t-tests. Statistical analyses were performed using the statistical software package JMP version 16 (SAS Institute Inc., Cary, NC, USA).

## Results

This study included a total of 321 students. The characteristics of the students in 2019 (*n* = 106), 2020 (*n* = 104), and 2021 (*n* = 111) are presented in Table 1. Out of them, 65 were female (20.2%), with a gradual increase in number from 2019 to 2021. In 2021, there was a higher percentage of female students, but no significant sex ratio difference was observed across the years. Additionally, the average (SD) English test scores of students on the university entrance exam were 183.7 (10.5) in 2019, 183.3 (6.5) in 2020, and 184.7 (9.9) in 2021, without any significant difference across the years.

**Table 1:** Characteristics of the study participants (*n* = 321)

Year	2019	2020	2021	*P*-value
Number	106	104	111	
Female, n (%)	19 (17.9)	19 (18.2)	27 (24.3)	0.413
National Center Test for University Admissions (English test score)*	183.7 ± 10.5	183.3 ± 6.5	184.7 ± 9.9	0.293

^*^mean ± standard deviation, out of 200

**Fig. 1:**
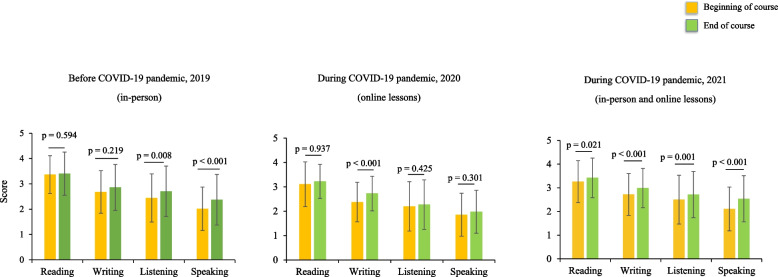
Subjective assessment of English skills before and during the COVID-19 pandemic. Self-report of reading, writing, listening and speaking skills on a Likert scale from 1 to 5. *P*-values were calculated using paired t-test. Data are presented as mean ± standard deviation

Throughout the study period, we found the highest self-estimated score for English reading skills, followed by writing, listening, and speaking skills (Fig. 1). We compared the improvements in these four general English skills at the beginning and end of the course for the same students using paired t-tests. Compared to the beginning of the course, we found improved self-assessed listening (*p* = 0.008) and speaking (*p* < 0.001) skills at the end of the course in 2019 using the in-person teaching method. However, we did not observe a significant increase in reading or writing skills. In 2020, when the course was held fully online, we have only observed a significant improvement in writing skills after the course (*p* < 0.001). We observed improvements in all skills in 2021 with combined in-person and online teaching, including reading (*p* = 0.021), writing (*p* < 0.001), listening (*p* = 0.001), and speaking (*p* < 0.001). Additionally, students attended online and combined courses more regularly than in-person courses (*p* ≤ 0.001) (Fig. S2).

**Fig. 2:**
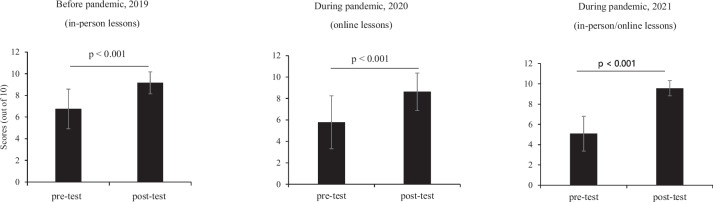
Objective assessment of knowledge of medical terminology using pre- and post-tests before and during the COVID-19 pandemic. *P*-values were calculated using paired t-test. Data are presented as mean ± standard deviation

**Fig. 3:**
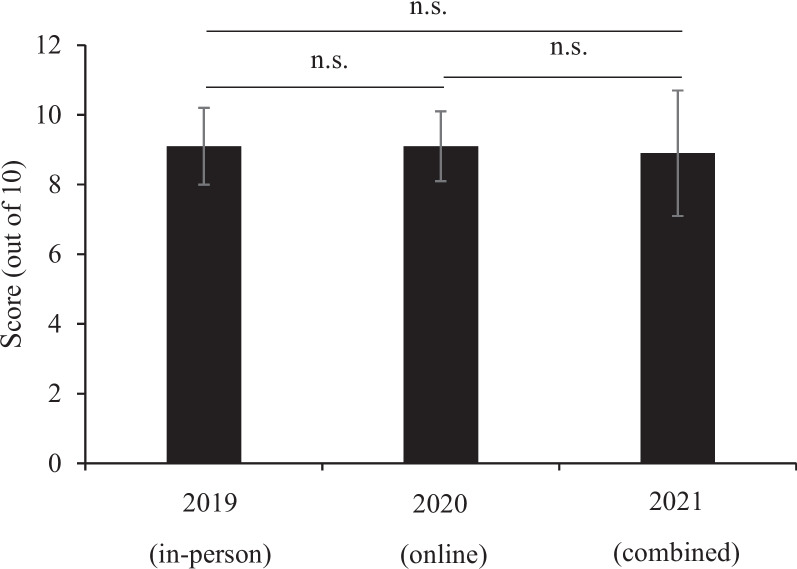
Influence of teaching styles on evidence-based medicine skills. n.s.: non-significant. Data are presented as mean ± standard deviation

**Fig. 4:**
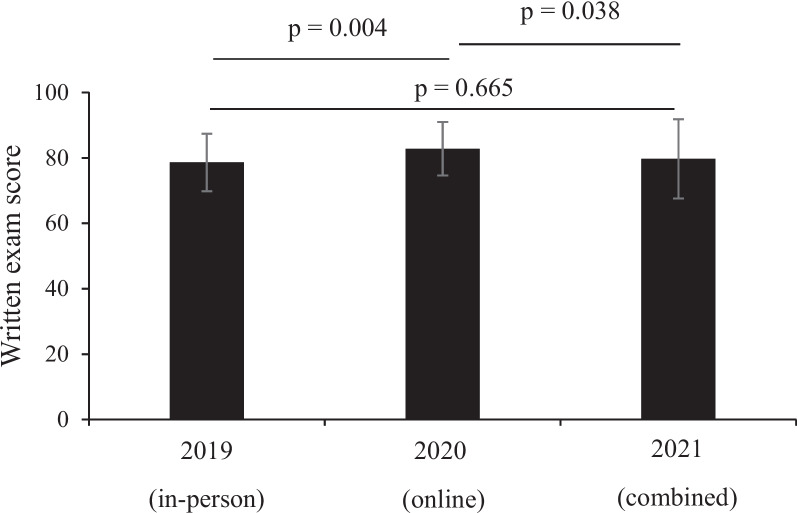
Influence of teaching styles on students’ final scores. Data are presented as mean ± standard deviation

In the assessment of medical terminology knowledge, we found a significant increase in scores in the post-test compared with the pre-test, regardless of the teaching style (*p* < 0.001) in each year (Fig. 2). We found similar scores in another objective assessment examining evidence-based medicine with in-person, online, and blended methods (Fig. 3), suggesting that teaching style did not differentially influence this outcome. The mean (SD) scores of the students in the final exam (out of 100 scores) were as follows: 78.6 (8.8) in 2019, 82.8 (8.2) in 2020, and 79.7 (12.1) in 2021. Students had significantly higher final exam scores in the online course than in the in-person (*p* = 0.004) and combined courses (*p* = 0.038). However, there was no significant difference in the final exam scores of the students in the combined and in-person courses (*p* = 0.665) (Fig. 4, Table S1).

## Discussion

We found that the online course did not adversely influence the academic performance or core competencies of medical students’ medical English education during preclinical clerkship, including medical terminology scores, evidence-based medicine skills, and final exam performance. These objective assessments showed that the outcomes in online courses were not inferior to those of in-person classes and that it provided even better results. English listening and speaking skills improved during in-person education. However, this trend was observed only for writing skills in online courses in 2020. Notably, we found improvements in all English skills, including reading, writing, listening, and speaking skills, in 2021, when the combination of online and in-person education was utilized.

We previously reported the effects of the omnibus-style medical English course on the self-assessed English skills of our students before the COVID-19 pandemic [9]. Between 2016 and 2018, we observed improvements in listening and speaking skills. We found similar results in 2019, confirming the influence of our course on listening and speaking skills during face-to-face lessons. Improvements in writing skills but not in listening and speaking skills for online courses could be explained by the lack of face-to-face communication with students, resulting in lower speaking and listening skills. By contrast, because of the greater focus on writing assignments in the online course, students had better writing skills after the online course. In 2021, when we combined in-person and online lessons, we found varied effects of both types of instruction on reading, writing, listening, and speaking skills. Taken together, the subjective assessment of such skills is highly influenced by the teaching style, and online or combined lessons may provide better outcomes.

In addition to the self-assessment survey, we conducted objective surveys to examine the influence of online education on medical students’ academic performance. The results from the pre-and post-tests for medical terminology showed that students gained scores regardless of the teaching method. Additionally, the scores for evidence-based medicine skills and the final exam did not show any differences between online, in-person education, or a combination of these. Yao et al. (2021) conducted pathophysiology teaching with online education resources and live video discussions in China, which did not influence the overall performance of medical students during the pandemic compared to traditional teaching plans. Notably, they observed an increase in the proportion of students with higher final test scores (> 90 points). In line with this, our students had higher final scores in online courses or in a combination of in-person and online courses than in traditional courses [7].

A recent systematic review and meta-analysis examined whether online versus offline learning influenced learning outcomes among undergraduate medical students [10]. The meta-analysis found that online learning was as effective as offline learning. Also, a randomized controlled trial (RCT) showed that computer-based teaching is as effective as in-person lecture-based teaching of evidence-based medicine at post-graduate levels [11]. Our results from objective surveys, which are consistent with this meta-analysis, suggest that online teaching is not inferior to in-person teaching. It could be an effective method in preclinical clerkship medical education in the future.

Online learning is a powerful tool in education; however, its application of online learning in medicine must be interpreted with caution. Previous studies have suggested the effectiveness and usefulness of online medical education [7, 12, 13]. However, the present data mainly focused on preclinical clerkship and knowledge and not on clinical skills during clinical clerkship. An observational study in Poland reported medical students’ perceptions of online learning during the pandemic; the students reported the main advantages of online learning: the ability to stay at home, flexible access to online materials, learning at their own pace, and comfortable surroundings [5]. Although there was no difference between in-person and online learning in improving knowledge, online learning was less effective than in-person learning in improving skills and social competencies. A systematic review [4] examined the effectiveness of online versus alternative training methods among licensed healthcare professionals and summarized the results for RCTs, excluding observational studies between 2000 and 2015. It found that online training, compared to delivered lectures, showed favorable results in terms of knowledge and practical skills; however, it did not reach the significance threshold. The authors concluded that the quality of evidence for all comparisons included in their meta-analysis was low or very low, without reaching certain conclusions. We also examined the influence of online training on knowledge but not on clinical skills in preclinical clerkships. Therefore, the effect of online programs on practical skills requires further high-quality research.

Several issues must be considered to achieve better outcomes in online teaching. Lack of internet access, technical problems, poor learning environments at home, and a lack of self-discipline are potential factors influencing the quality and outcomes of online courses. Additionally, decreased student engagement in discussions, lack of opportunity to ask questions and involve in teamwork, and lack of interaction with patients and other students, along with mental health problems, are further concerning points [5, 14]. On the mentor’s side, limitations regarding the creation of interactive materials and knowledge of the latest technology and online learning modalities are challenging [15, 16]. Therefore, investments in digital infrastructure, training staff, and faculty members are necessary to create high-quality, interactive online courses that engage learners effectively and support students with the development of a safe-learning environment [17, 18]. Ultimately, blended courses and using on-demand materials in addition to synchronized distance education may significantly improve the quality of medical education in the future [19]. In the current analysis, we observed higher student attendance in online and combined courses compared to in-person courses. This may partially explain the better performance and outcomes in online and combined courses compared to in-person courses. Further comprehensive studies are warranted to conduct and focus on the underlying factors in the online/combined courses compared to traditional courses that lead to better outcomes.

Before the COVID-19 pandemic, we reported an increasing trend in outbound student mobility among Japanese medical students participating in short-term exchange programs, taking the United States Medical Licensing Examination (USMLE), engaging in clinical training, and undertaking research abroad between 2016 and 2019 [9, 20]. About 67.8% of the students wished to engage in at least one of these outbound activities in 2019; however, this declined to half in 2020 during the pandemic. This indicates the adverse influence of the pandemic on medical students’ plans to go abroad for clinical and research training [19]. In a follow-up study, we found a promising and significant increase in the number of students studying and training abroad in 2021 compared to 2020, which is close to the pre-pandemic level [21]. We hope that such an increase in the outbound mobility of Japanese medical students, along with lessons learned during the pandemic and online teaching, will help us move medical education to a higher level and nurture the next generation of physicians. Additionally, communicating and working with authorities within and outside universities to prevent, mitigate, and respond to future pandemics is warranted.

This study had an acceptable sample size with a high response rate (approximately 97.5%). The data were collected over three consecutive years with comparable baseline characteristics of the students, such as sex ratio and university entrance exam English scores; however, we cannot rule out the possibility of some bias in this analysis because other sociodemographic characteristics were not captured in this study. Therefore, we compared our findings from 2019 with those from 2016 to 2018, before the COVID-19 pandemic, using the same teaching style. We found similar results, suggesting that the findings in 2019 are probably not accidental and are appropriate for comparison with the results in 2020 and 2021. Additionally, we conducted an institutional survey and found no net access limitations or technical problems in participating in the online course (data not shown), suggesting that these factors did not influence the current results significantly. However, this study has several limitations. This study focused mainly on medical students’ knowledge and not on their clinical skills. There is a possibility that unmeasured covariates and socio-demographic characteristics may have influenced the results. In addition, the current analysis was conducted in one course, and this course is not representative of all courses in preclinical medical education. Future large-scale studies focusing on more preclinical courses among medical students are warranted. In addition, the results of this study need to be replicated in future studies with longitudinal follow-up in more countries with different educational systems and cultures than Asian countries.

## Conclusions

This study found that, during the COVID-19 pandemic, online/combined education was effective and not inferior to in-person education. The educational outcome was examined by implementing subjective and objective assessments of medical students during their preclinical clerkship. The development of online/combined courses in the medical school curriculum during this period is therefore seen as promising. Well-designed outcome-oriented online/combined courses can improve different domains of learning even after the pandemic and serve as alternatives in the event of another unprecedented situation in the future.

### Supplementary Information

**Additional file 1:** **Figure S1.** Number of active cases and mortality of COVID-19 in Japan during conducting the current study. **Figure S2.** Attendance scores. **Table S1.** Students’ final exam scores before and during the pandemic.

## Data Availability

The datasets analyzed during the current study are available in the Figshare repository as follows: Goudarzi, Houman; Onozawa, Masahiro; Takahashi, Makoto (2022). Impact of Covid-19 pandemic and consequent online teaching on medical education: subjective and objective assessment. figshare. Dataset. 10.6084/m9.figshare.19736443.v1.
