# Supplementary material for: Impact of the Covid-19 pandemic and ensuing online teaching on pre-clinical medical education
Source: BMC Med Educ. 2024 Jan 17;24:66. doi: 10.1186/s12909-023-04967-x (PMC10792807; doi:10.1186/s12909-023-04967-x)
Supplement: Supplementary file 1 — Additional file 1: Figure S1. Number of active cases and mortality of COVID-19 in Japan during conducting the current study. Figure S2. Attendance scores. Table S1. Students’ final exam scores before and during the pandemic. [file 12909_2023_4967_MOESM1_ESM.docx]

**Impact of the Covid-19 pandemic and ensuing online teaching on**

**pre-clinical medical education**

Houman Goudarzi^1^, Masahiro Onozawa^2^, Makoto Takahashi^1^

^1^Center for Medical Education and International Relations, Faculty of Medicine and

Graduate School of Medicine, Hokkaido University, Japan

^2^Clinical Training Center, Hokkaido University Hospital, Japan

**Corresponding Author**: Makoto Takahashi. M.D., Ph.D.

Center for Medical Education and International Relations, Faculty of Medicine and Graduate School of Medicine, Hokkaido University, Sapporo, Japan

E-mail: mtakahashi@pop.med.hokudai.ac.jp

Fax: +81-11-706-7628; Phone: +81-11-706-5547

ORCiDs

Houman Goudarzi: 0000-0003-1145-8917

Masahiro Onozawa: 0000-0001-9267-2864

Makoto Takahashi: 0000-0001-5810-1224

**Real-time changes in our courses regarding COVID-19 measures**

Before the pandemic, our course was conducted fully in-person between April and July 2019. The COVID-19 pandemic was declared in March 2020, and our university switched all teaching programs in pre-clinical clerkship to online. Therefore, all second-year students who took courses online in 2020 did not come to classrooms or use university facilities except to take the final exam. In 2021, we adjusted teaching styles according to the Business Continuity Plan (BCP) level announced by our university, accessible from the following link:

<https://www.global.hokudai.ac.jp/wp-content/uploads/2020/04/2020.4.16-BCP-during-Coronavirus-Outbreak.pdf>

We started the course in April 2021 in-person and switched to online in May and June, then switched back to in-person in July. We briefly showed the number of cases and mortality in Japan in the following figure to illustrate the change in teaching styles during our courses.

**Content of the course**

The details of the lessons in our courses are listed below:

1) Orientation/survey (1 session)

2) Basic rules and principles of medical terminology (3 sessions)

3) Medical communication in clinical settings (2 sessions)

4) Career development (2 sessions)

5) Common medical terminology in medicine (1 session)

6) Common clinical case scenarios in primary care (1 session)

7) EBM skills and academic reading practice of scientific papers (2 sessions)

8) Differences in healthcare systems between Japan and other countries (1 session)

9) Skills for scientific presentations/key features of academic writing (2 sessions)

10) Survey, final exam (1 session)

Regarding learning strategies, we applied active learning for medical communication in clinical settings, EBM skills, and skills for scientific presentations (a total of 6 lessons).

Figure S1. Number of active cases and mortality of COVID-19 in Japan during conducting the current study.


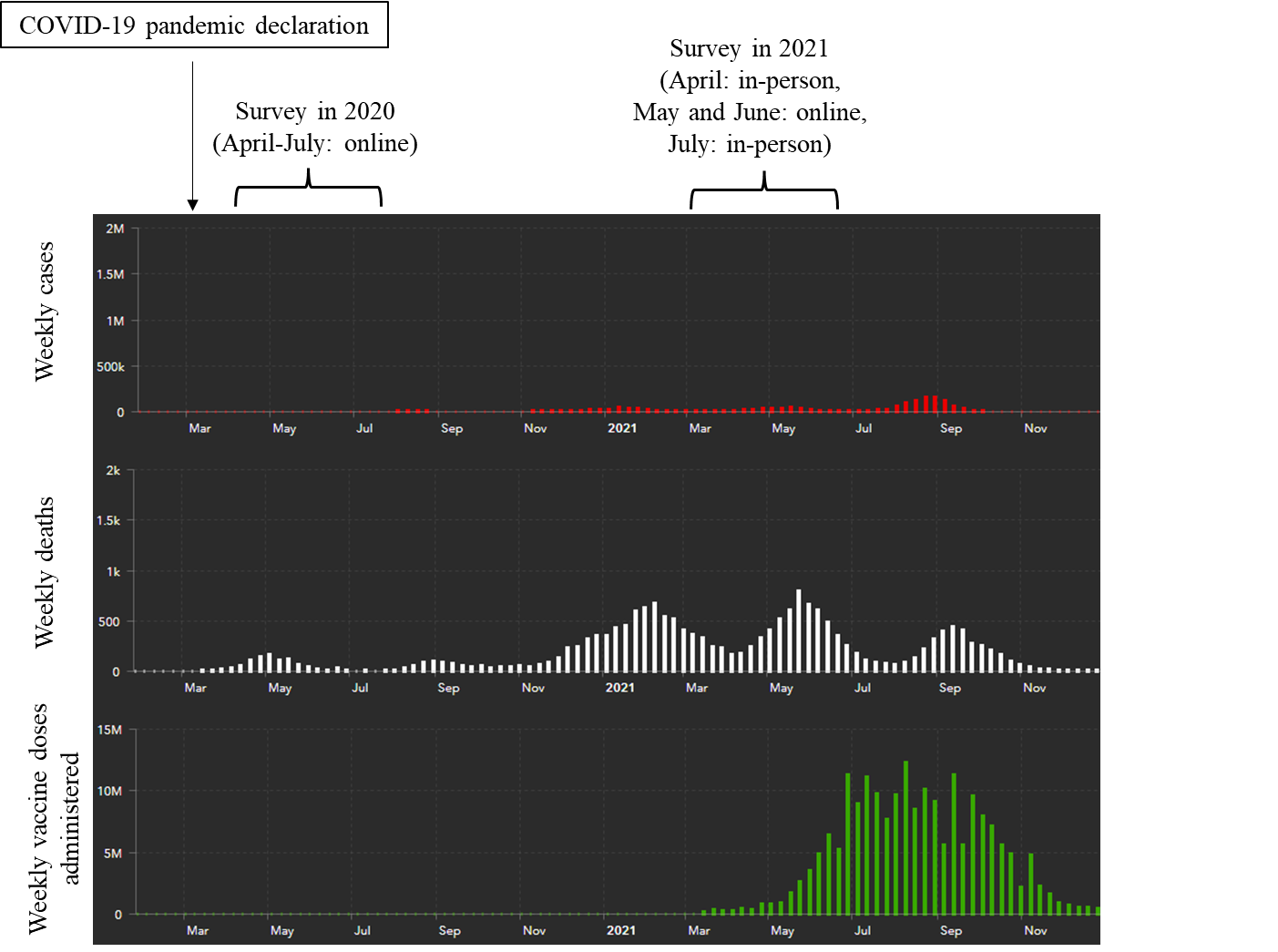


Data from The Johns Hopkins Coronavirus Resource Center

<https://coronavirus.jhu.edu/map.html>

Figure S2. Attendance scores.

2021

(In-person and online)

2020

(Online)

2019

(In-person)

A maximum of 25 scores

Table S1. Students’ final exam scores before and during the pandemic.

| **Year** | **10%tile** | **25%tile** | **median** | **75%tile** | **90%tile** | **Mean** | **SD** |
| --- | --- | --- | --- | --- | --- | --- | --- |
| 2019 | 64 | 72 | 80 | 84 | 88.4 | 78.6 | 8.8 |
| 2020 | 72 | 76 | 84 | 88 | 92 | 82.8 | 8.2 |
| 2021 | 60.4 | 72 | 80 | 88 | 95.6 | 79.7 | 12.1 |
